# Enhanced Thermoelectric Properties of Nb-Doped Ti(FeCoNi)Sb Pseudo-Ternary Half-Heusler Alloys Prepared Using the Microwave Method

**DOI:** 10.3390/ma16165528

**Published:** 2023-08-09

**Authors:** Ruipeng Zhang, Jianbiao Kong, Yangbo Hou, Linghao Zhao, Junliang Zhu, Changcun Li, Degang Zhao

**Affiliations:** 1 School of Materials Science and Engineering, University of Jinan, Jinan 250022, China; 2 Heze Institute of Product Inspection and Testing, Heze 274000, China; 3 Shenzhen Institute of Advanced Electronic Materials, Shenzhen Institute of Advanced Technology, Chinese Academy of Sciences, Shenzhen 518055, China

**Keywords:** thermoelectric, half-Heusler, pseudo-ternary

## Abstract

Pseudo-ternary half-Heusler thermoelectric materials, which are formed by filling the B sites of traditional ternary half-Heusler thermoelectric materials of ABX with equal atomic proportions of various elements, have attracted more and more attention due to their lower intrinsic lattice thermal conductivity. High-purity and relatively dense Ti_1−*x*_Nb*_x_*(FeCoNi)Sb (*x* = 0, 0.01, 0.03, 0.05, 0.07 and 0.1) alloys were prepared via microwave synthesis combined with rapid hot-pressing sintering, and their thermoelectric properties are investigated in this work. The Seebeck coefficient was markedly increased via Nb substitution at Ti sites, which resulted in the optimized power factor of 1.45 μWcm^−1^K^−2^ for *n*-type Ti_0.93_Nb_0.07_(FeCoNi)Sb at 750 K. In addition, the lattice thermal conductivity was largely decreased due to the increase in phonon scattering caused by point defects, mass fluctuation and strain fluctuation introduced by Nb-doping. At 750 K, the lattice thermal conductivity of Ti_0.97_Nb_0.03_(FeCoNi)Sb is 2.37 Wm^−1^K^−1^, which is 55% and 23% lower than that of TiCoSb and Ti(FeCoNi)Sb, respectively. Compared with TiCoSb, the *ZT* of the Ti_1−*x*_Nb*_x_*(FeCoNi)Sb samples were significantly increased. The average *ZT* values of the Nb-doped pseudo-ternary half-Heusler samples were dozens of times that of the TiCoSb prepared using the same process.

## 1. Introduction

The non-renewable nature of traditional mineral energy and the environmental pollution caused by its consumption pose serious challenges to the energy supply of global economic development [1,2]. Thermoelectric materials, as a kind of green energy material that can directly convert heat and electricity into each other, have great potential in waste heat utilization and thermoelectric refrigeration, which can make great contributions to solve the energy crisis [3,4,5]. In the history of materials science, a variety of performance parameters are used in order to quantify the merits of a material more intuitively. The quantitative parameter used to measure the performance of thermoelectric materials is the dimensionless figure of merit *ZT* as follows:

(1) $$ZT=S^{2}\sigma T/\kappa$$

where *S^2^σ* is the power factor (*PF*), obtained from the Seebeck coefficient (*S*) and electrical conductivity (*σ*), *T* is the absolute temperature and *κ* is the thermal conductivity, which is the sum of the carrier thermal conductivity (*κ_E_*) and lattice thermal conductivity (*κ_L_*) [6,7]. It can be seen from Formula (1) that the enhancement of performance usually starts from the numerator and denominator aspects. The former is to increase the *PF* by means of non-equivalent atom doping [8,9,10], energy band engineering, etc. [11,12,13], while the latter often uses nano engineering [14,15,16], defect engineering and other means to reduce the lattice thermal conductivity [17,18]. Due to the coupling relationship between the parameters, an excessive pursuit of the optimization of a single parameter often cannot achieve the enhancement of the *ZT* of thermoelectric materials [19]. For example, an excessive pursuit of a large carrier concentration to increase the conductivity can inevitably lead to a deterioration of the Seebeck coefficient. Therefore, how to achieve a greater optimization of performance remains a crucial part of thermoelectric research.

The half-Heusler (HH) compound crystallizes into the cubic MgAgAs-type structure, with ABX as the general chemical formula, which should be viewed as the interpenetration of four face-centered cubic lattices. Elements at the X position are usually elements of the main group, while elements at the A and B positions are elements of the transition group [20]. The elements at the A, B and X positions of the HH can be replaced by different elements without destroying their crystal structure. Half-Heusler alloys are superior thermoelectric materials at the middle and high temperature region due to their excellent electrical conductivity and Seebeck coefficient brought about by their highly symmetric crystal structures [20,21]. In addition, their better thermal stability and mechanical properties compared with other types of thermoelectric materials are also indispensable factors [22]. However, the high symmetry of the crystal structure makes HH compounds not only have excellent electrical properties, but also a high thermal conductivity, which is unfavorable to *ZT* according to the formula. The excessive thermal conductivity of traditional ternary HH thermoelectric materials has become the limit of their commercial application, and researchers have tried various optimization strategies to reduce their thermal conductivity [23,24,25]. The lattice thermal conductivity, which is the main contribution of *κ*, is largely determined by the number of atoms in the primitive unit cell (*N*) [26]. The strategy of reducing the thermal conductivity by increasing *N* to introduce more disorder and lattice distortion based on the high-entropy core effect has been proven in many thermoelectric material systems such as Ge_0.61_Ag_0.11_Sb_0.13_Pb_0.12_Bi_0.01_Te [27], AgSnSbSe_3_ [28], BiSbTe_1.5_Se_1.5_ [29], Sn_0.25_Pb_0.25_Mn_0.25_Ge_0.25_Te [30], etc. The high substitutability of atoms at each position of HH compounds fits well with this strategy. Consequently, pseudo-ternary half-Heusler thermoelectric materials (*N* > 3) with a low intrinsic lattice thermal conductivity caused by disorder scattering and a smaller phonon group velocity have attracted widespread attention in recent years [31]. And many encouraging pseudo-ternary HH results have been achieved [32]. Wang et al. [33] prepared the Ti_2_FeNiSb_2_ double half-Heusler compound by increasing the number of atoms in the primitive unit cell. On the basis of replacing Sb with Sn, the small lattice thermal conductivity of 1.95 Wm^−1^K^−1^ and the peak *ZT* value of 0.52 for Ti_1.6_Hf_0.4_FeNiSb_1.7_Sn_0.3_ are obtained at 923 K. Luo et al. [34] and Wang et al. [35] realized the *p*-*n* transformation and the thermoelectric performance optimization of Ti(Fe_1/3_Co_1/3_Ni_1/3_)Sb by adjusting the ratio of Fe and Ni.

It is commendable that, as verified by previous reports, microwave-synthesized samples have the advantages of refining grains and improving the uniformity of composition [17,36,37,38,39]. With the development in recent years, the technique of microwave synthesis has been relatively reliable, and the dense bulk ceramic or metallic samples can be synthesized in a few minutes with the help of carbon, silicon carbide and other dielectric materials with a high dielectric constant, which greatly reduces the preparation cycle and cost [40,41,42]. Birkel et al. [43] successfully prepared TiNiSn half-Heusler compounds by using 3.6 g of granular carbon as an absorbing material via microwave heating for 1 min. Its performance was tested and compared with that of TiNiSn prepared using the arc melting method. The thermoelectric properties of the two samples are basically the same except for the presence of a trace impurity phase. Lei et al. [44] also successfully synthesized a TiNiSn half-Heusler compound by heating it in a microwave device for 5 min. In addition, excellent thermoelectric material systems such as Mg_2_Si [45], PbTe [46] and Bi_2_Te_3_ [47] have been documented to prove the successful application of a microwave in their preparation. In this work, high-purity and high-density Ti_1−*x*_Nb*_x_*(FeCoNi)Sb (*x* = 0, 0.01, 0.03, 0.05, 0.07 and 0.1) pseudo-ternary half-Heusler alloys were prepared via microwave synthesis combined with the ball-milling and rapid hot-pressing sintering processes, and the effects of Nb substitution at the Ti site on its phase composition, microstructure and thermoelectric properties were studied. As a comparison, a TiCoSb sample was prepared using the same technical route. This work is expected to provide a feasible idea for optimizing the properties of pseudo-ternary HH thermoelectric materials with a more economical synthesis process.

## 2. Materials and Methods

**Figure 1:**
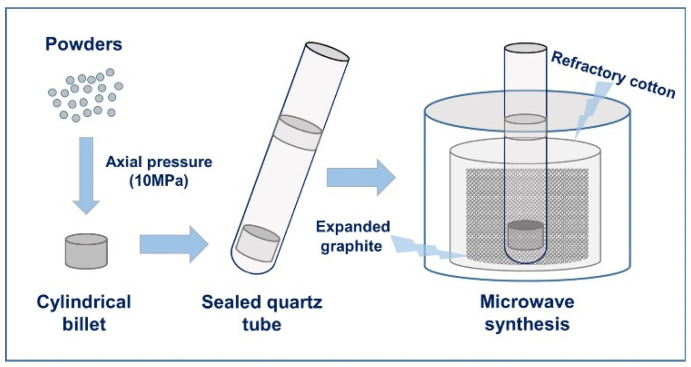
The process of microwave synthesis.

The original powders of TiCoSb and Ti_1−*x*_Nb*_x_*(FeCoNi)Sb (*x* = 0, 0.01, 0.03, 0.05, 0.07 and 0.1) samples were titanium powder (99.98%, 300 mesh, MACKLIN, Shanghai, China), niobium powder (99.999%, 300 mesh, MACKLIN, Shanghai, China), iron powder (99.9%, 200 mesh, MACKLIN, Shanghai, China), cobalt powder (99.8%, 200 mesh, MACKLIN, Shanghai, China), nickel powder (99.99%, 200 mesh, MACKLIN, Shanghai, China) and antimony powder (99.9%, 800 mesh, MACKLIN, Shanghai, China). The powders, which were weighed in accordance with stoichiometric ratio, were evenly mixed and then loaded into a cold-pressed mold, which was pressed under an axial pressure of 10 MPa for 5 min to obtain cylindrical billet at room temperature. Then, the obtained billet was put into the prepared clean quartz tube for vacuum sealing (≤0.01 Pa) to ensure that the billet would not oxidize during the synthesis process. The sealed quartz tube was placed in an alumina crucible that filled with expanded graphite powder (99.9%, 10–30 μm, MACKLIN, Shanghai, China) as an absorbing material. The whole tube was placed in a self-made microwave apparatus for microwave synthesis for 5 min. The sketch map of the microwave synthesis device is shown in Figure 1, and its power is 900 W. Then, the block billet obtained via microwave synthesis was mechanically broken and ground for 5 h in a planetary ball mill at a rotating speed of 300 rpm. The compact disc samples were obtained by loading the ball-milled powder in batches into a hot pressing mold with a diameter of 12 mm, and rapid hot-pressing sintering for 20 min at a temperature of 1073 K with a pressure of 80 MPa. In the process of rapid hot-pressing sintering, the heating rate was 100 K/min.

Ti_1−*x*_Nb*_x_*(FeCoNi)Sb (*x* = 0, 0.01, 0.03, 0.05, 0.07 and 0.1) alloys were characterized via X-ray diffraction (XRD) using a Rigaku Ultima IV diffractometer in the 2*θ* range of 10–90°. The surface morphology and microstructure of samples were analyzed using a scanning electron microscope (SEM, JEOL, JXA-8100, Tokyo, Japan) and energy dispersive spectrometer. The Archimedes method was used to measure the density of Ti_1−*x*_Nb*_x_*(FeCoNi)Sb alloys. The electrical conductivity and Seebeck coefficient of alloys at room temperature to 750 K can be measured simultaneously using the ZEM-3 system (ULVAC-RIKO, Yokohama, Japan) in a low-pressure (~10^2^ Pa) helium atmosphere. The thermal conductivity (*κ*) of samples can be calculated according to the formula $\kappa =C_{P}d\lambda$, where *C_p_* is the specific heat capacity of material, *d* is the density and *λ* is the thermal diffusion coefficient. The thermal diffusivity coefficient of Ti_1−*x*_Nb*_x_*(FeCoNi)Sb alloys can be obtained using the laser flash thermal analyzer (LFA-457, Netzsch, Bavaria, Germany). The estimated error of measurement of the above physical parameters (*d*, *σ*, *S* and *κ*) was at most 5%.

## 3. Results and Discussion

### 3.1. Phase Analysis

As shown in Table 1, the relative densities of the Ti_1−*x*_Nb*_x_*(FeCoNi)Sb (*x* = 0, 0.01, 0.03, 0.05, 0.07 and 0.1) alloys are above 95%, which were measured over more than three measurements. A relatively high density is usually positively correlated with the electrical properties of thermoelectric materials [48].

**Figure 2:**
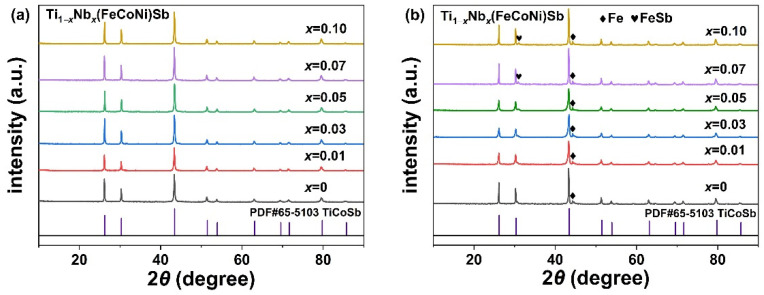
The XRD spectra of powder samples after microwave synthesis (**a**) and compact disc samples after rapid hot-pressing sintering (**b**) of Ti_1−*x*_Nb*_x_*(FeCoNi)Sb (*x* = 0, 0.01, 0.03, 0.05, 0.07 and 0.1) alloys.

Figure 2 shows the X-ray diffraction results of the Ti_1−*x*_Nb*_x_*(FeCoNi)Sb (*x* = 0, 0.01, 0.03, 0.05, 0.07 and 0.1) pseudo-ternary half-Heusler alloys prepared via microwave synthesis and rapid hot-pressing sintering. The main diffraction peaks of the samples were well indexed to the cubic half-Heusler TiCoSb (PDF#65-5103), which indicated that the pseudo-ternary HH thermoelectric materials with a high density can be successfully prepared via microwave synthesis combined with rapid hot-pressing sintering. As can be seen from Figure 2a, the XRD spectrum of the powder showed no other impurity phase except for the half-Heusler phase. However, the XRD results of the sintered samples in Figure 2b show the presence of trace Fe and FeSb secondary phases. The phase fractions of the Ti_1−*x*_Nb*_x_*(FeCoNi)Sb (*x* = 0, 0.01, 0.03, 0.05, 0.07 and 0.1) alloys are shown in Table 2. The existence of these metallic secondary phases inevitably affected the electrical conductivity and thermal conductivity of the Ti_1−*x*_Nb*_x_*(FeCoNi)Sb alloys [23,49,50,51,52,53].

### 3.2. Microstructural Characterization

**Figure 3:**
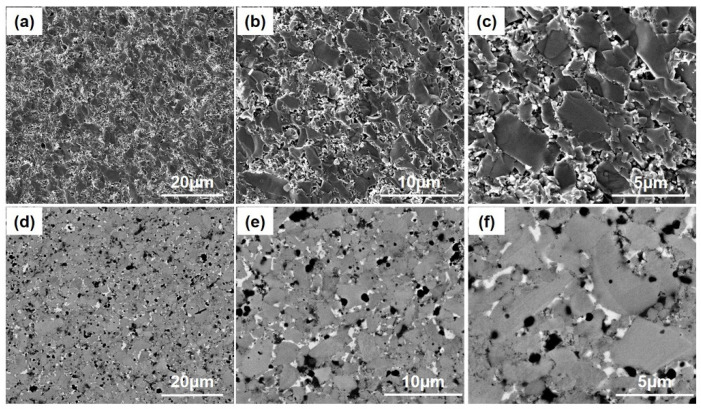
The fractural cross-section secondary electron images (**a**–**c**) and back-scattering electron images (**d**–**f**) of the Ti_0.9_Nb_0.1_(FeCoNi)Sb sample at different magnifications.

**Figure 4:**
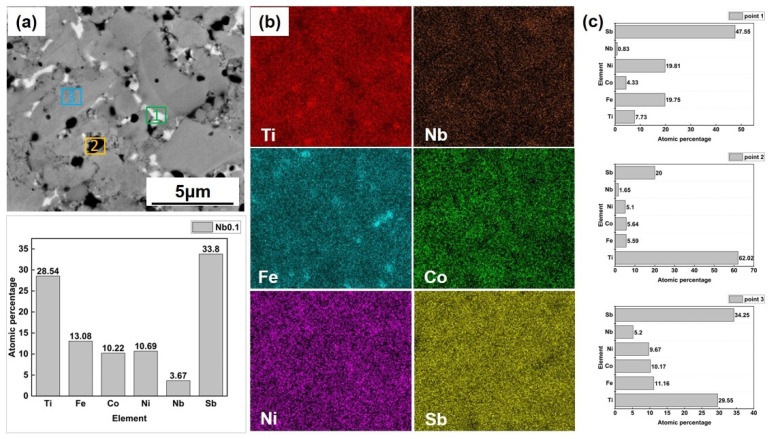
The EDX compositional surface analysis (**a**), mapping (**b**) and point analysis (**c**) of the Ti_0.9_Nb_0.1_(FeCoNi)Sb sample.

The fractural cross-section secondary electron images of the Ti_0.9_Nb_0.1_(FeCoNi)Sb sample in Figure 3a–c show the existence of small grains among large grains. In addition, the back-scattering electron images of the Ti_0.9_Nb_0.1_(FeCoNi)Sb sample also show obvious phase segregation, just as shown in Figure 3d–f. To determine the phase composition, the energy dispersive X-ray (EDX) compositional point analysis and compositional mapping analysis were carried out on the Ti_0.9_Nb_0.1_(FeCoNi)Sb sample. As shown in Figure 4a, the atomic ratio of Fe, Co and Ni obtained via the EDX compositional surface analysis was close to 1:1:1. Moreover, the mapping results of the Ti_0.9_Nb_0.1_(FeCoNi)Sb sample show the constituent element segregation, which verifies the presence of the secondary phases in the XRD results. Based on the results of the compositional point analysis, it can be speculated that the gray regions (point 3, blue) were the Ti_0.9_Nb_0.1_(FeCoNi)Sb HH matrix phase, the dark regions (point 2, yellow) were the Ti-rich phase and Fe-rich phase and the bright regions (point 1, green) were the Sb-rich phase.

### 3.3. Thermoelectric Transport Properties

**Figure 5:**
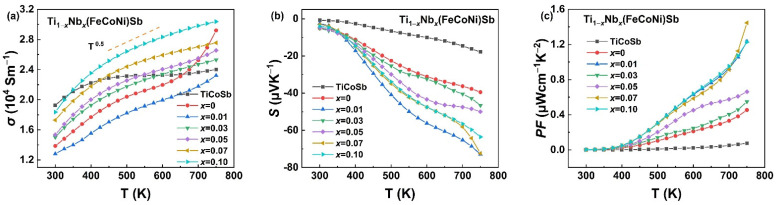
The curves of the electrical properties of TiCoSb and Ti_1−*x*_Nb*_x_*(FeCoNi)Sb (*x* = 0, 0.01, 0.03, 0.05, 0.07 and 0.1) alloys. Electrical conductivity (**a**), Seebeck coefficient (**b**) and power factor (**c**).

The electrical properties of the Ti_1−*x*_Nb*_x_*(FeCoNi)Sb (*x* = 0, 0.01, 0.03, 0.05, 0.07 and 0.1) alloys as a function of temperature are shown in Figure 5. Except for the TiCoSb and Ti(FeCoNi)Sb samples, the *σ* of all the samples followed a temperature dependence of *T*^0.5^, implying that the alloy disorder scattering and ionized impurity scattering caused by the Ti-Nb heteroatomic substitution dominated the charge transport [54,55]. As shown in Figure 5a, except for the decrease in the conductivity of Ti_0.99_Nb_0.01_(FeCoNi)Sb, the electrical conductivity of the other samples increased due to the addition of valence electrons involved in electrical transport [20,56]. The decrease in Ti_0.99_Nb_0.01_(FeCoNi)Sb may be due to the fact that although the incorporation of Nb led to an increase in the carrier concentration (*n*), it was not enough to compensate for the decrease in the carrier mobility that arose from the introduction of point defects. However, when *x* > 0.01, with the increase in carrier concentration, the adverse impact of ionized impurity scattering on the carrier mobility was weakened due to the screening effect [57]. In addition, the presence of metallic secondary phases constructed the internal high-speed channel of electrons inside the samples, which improved the electrical conductivity.

**Figure 6:**
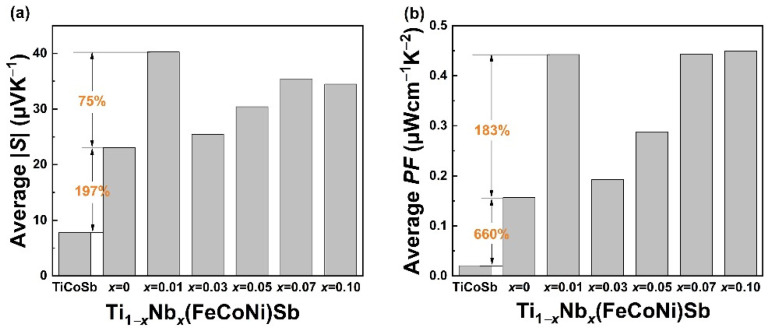
The average Seebeck coefficient (300–750 K) (**a**) and average power factor (300–750 K) (**b**) of TiCoSb and Ti_1−*x*_Nb*_x_*(FeCoNi)Sb (*x* = 0, 0.01, 0.03, 0.05, 0.07 and 0.1) alloys.

Figure 5b shows that the Seebeck coefficient was negative in the range of 300 K to 750 K, indicating an *n*-type conducting behavior of the Ti_1−*x*_Nb*_x_*(FeCoNi)Sb samples. For degenerate semiconductors, the Seebeck coefficient *S* is usually associated with the carrier concentration that can be represented by the Mott equation [19],

(2) $$S=\frac{8\pi^{2}{k_{B}}^{2}}{3eh^{2}}m^{*}T{\left(\frac{\pi }{3n}\right)}^{\frac{2}{3}}$$

where *k_B_*, *e*, *h*, *T*, m* and *n* are the Boltzmann constant, elementary charge, Planck constant, absolute temperature, density of states (DOSs), effective mass and carrier concentration, respectively. As shown in Figure 6a, the absolute values of *S* and the average values of |*S*| of all the Nb-doped samples were larger than that of TiCoSb and Ti(FeCoNi)Sb, indicating that the incorporation of Nb can be beneficial to the improvement of the Seebeck coefficient, which can be due to the increase in the effective mass [34]. However, it can be seen from formula (2) and Table 1 that as the Nb content increased, the coupling relationship between the carrier concentration and the Seebeck coefficient hindered the increase in the Seebeck coefficient [58]. A large m* of Ti_1−*x*_Nb*_x_*(FeCoNi)Sb is conducive to the high Seebeck coefficient, but may, in turn, lead to a deterioration in the carrier mobility (*μ*) if the band effective mass is also high [59]. Furthermore, the intensified alloy scattering of carriers resulted from the Nb doping was also unfavorable for *μ*. The power factors (*PFs*) calculated using the electrical conductivity and Seebeck coefficient of the Ti_1−*x*_Nb*_x_*(FeCoNi)Sb alloys are shown in Figure 5c. The *PF*s of all the pseudo-ternary alloys including the undoped Ti(FeCoNi)Sb were significantly higher than that of the TiCoSb alloy. The maximum *PF* value was obtained for Ti_0.93_Nb_0.07_(FeCoNi)Sb at 750 K, which was 1.45 μWcm^−1^K^−2^, which is much higher than that of Ti(FeCoNi)Sb. The average power factor of the Nb-doped Ti_1−*x*_Nb*_x_*(FeCoNi)Sb samples in Figure 6b also show significant improvement compared with TiCoSb and Ti(FeCoNi)Sb. It is worth noting that compared with other traditional tens or even hundreds of μWcm^−1^K^−2^ of power factor such as TiCoSb [60], TiNiSn [61] and NbFeSb [50], the result is not enough to support the development of the most outstanding HH thermoelectric materials. Consequently, it is still necessary to further explore the optimization of the electrical properties of the Ti(FeCoNi)Sb pseudo-ternary half-Heusler alloy.

**Figure 7:**
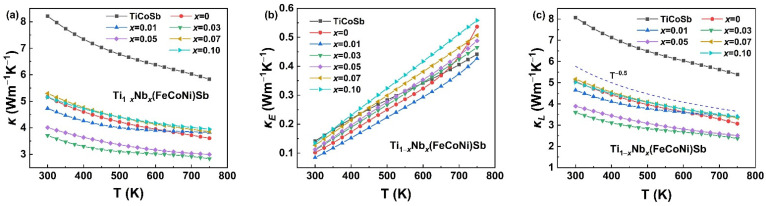
The curves of the thermal properties of TiCoSb and Ti_1−*x*_Nb*_x_*(FeCoNi)Sb (*x* = 0, 0.01, 0.03, 0.05, 0.07 and 0.1) alloys. Total thermal conductivity (**a**), electronic thermal conductivity (**b**) and lattice thermal conductivity (**c**).

The curves of the total thermal conductivity (*κ*), electronic thermal conductivity (*κ_E_*) and lattice thermal conductivity (*κ_L_*) of the Ti_1−*x*_Nb*_x_*(FeCoNi)Sb (*x* = 0, 0.01, 0.03, 0.05, 0.07 and 0.1) alloys with temperature are shown in Figure 7. The thermal conductivity was significantly reduced with the equal proportional substitution of Fe, Co and Ni at the Co sites over the measured temperature. The electronic thermal conductivity was calculated using the Wiedemann–Franz law as follows:

(3) $$\kappa_{E}=L\sigma T$$

where *L* is the Lorenz number. It can be seen from Figure 7b that the variation trend of the electronic thermal conductivity turned out to be consistent with that of the electrical conductivity, and there was no obvious gap between the samples. In contrast to the less noticeable drop in the electronic thermal conductivity, the decreasing trend of *κ* mainly resulted from the decrease in *κ_L_*. The lattice thermal conductivity *κ_L_* was obtained by subtracting the electronic contribution *κ_E_* from the total thermal conductivity *κ*. The lattice thermal conductivity of the Ti_1−*x*_Nb*_x_*(FeCoNi)Sb samples decreased with the increasing temperature, but this trend was partially slowed down at a high temperature due to the bipolar effect [62]. It can be observed from Figure 7c that the lattice thermal conductivity followed the *T*^−0.5^ dependence, implying that the alloy disorder scattering of phonons should be dominant [54]. In addition, the contribution of the ionized impurity scattering of phonons cannot be ignored [55,63]. For most HH materials, the simple crystal structure tends to result in a relatively high lattice thermal conductivity (≈10 Wm^−1^K^−1^ at 300 K) [64], which is more pronounced if a metallic phase is also present in the matrix [65]. Benefiting from the smaller group velocity phonons and disorder scattering caused by a more complex crystal chemistry [31], Ti(FeCoNi)Sb (5.05 Wm^−1^K^−1^ at 300 K) had the obviously lower *κ_L_* compared with that of TiCoSb (8.07 Wm^−1^K^−1^ at 300 K). The enhanced phonon scattering mechanism was attributed to several factors, such as point defects introduced by the substitution of Nb at the Ti sites, the mass and strain fluctuation and the lattice distortion generated by the increase in the configuration entropy with the equal proportional substitution of Fe, Co and Ni at the Co sites. Thus, the lattice thermal conductivity of the Nb-doped Ti_1−*x*_Nb*_x_*(FeCoNi)Sb alloys was lower than that of Ti(FeCoNi)Sb. However, when Nb > 0.05, this trend was inhibited under the influence of more metallic secondary phases. In the measured temperature range, the lattice thermal conductivity of Ti_0.97_Nb_0.03_MSb was decreased by 55% and 23% compared with that of TiCoSb and Ti(FeCoNi)Sb at the same temperature, respectively.

**Figure 8:**
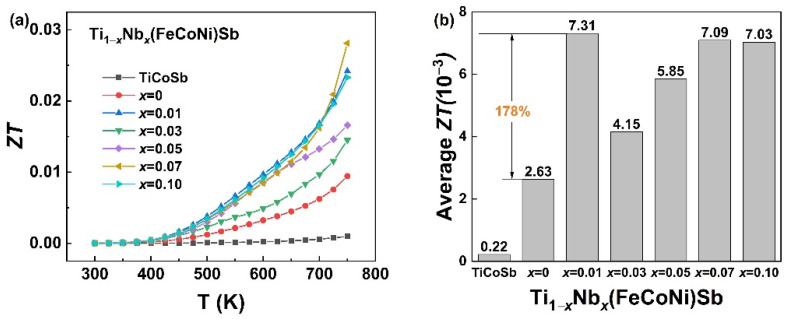
Temperature dependence of *ZT* (**a**) and average *ZT* (300–750 K) (**b**) of TiCoSb and Ti_1−*x*_Nb*_x_*(FeCoNi)Sb (*x* = 0, 0.01, 0.03, 0.05, 0.07 and 0.1) alloys.

Figure 8a,b shows the temperature dependence of the *ZT* and average *ZT* values (300–750 K) of the Ti_1−*x*_Nb*_x_*(FeCoNi)Sb (*x* = 0, 0.01, 0.03, 0.05, 0.07 and 0.1) alloys. With the increase in temperature, the *ZT* of each sample increased, but the gap also widened rapidly. The Nb doping and multi-element substitution at the Co sites resulted in a large gap in the *S* and *κ_L_* between samples. The maximum *ZT* value was about 0.03 for the Ti_0.93_Nb_0.07_(FeCoNi)Sb sample. However, this is still lower than the *ZTs* of the *p*-type Ti(Fe_1/3+0.15_Co_1/3_Ni_1/3−0.15_)Sb and *n*-type Ti(Fe_1/3−0.1_Co_1/3_Ni_1/3+0.1_)Sb at 750 K (above 0.2) reported by Luo et al. [34], which is speculated to be due to excessive differences in the electrical properties. Compared with the TiCoSb sample prepared using the same process, the *ZT* of the Ti_1−*x*_Nb*_x_*(FeCoNi)Sb samples were obviously improved due to the increase in the Seebeck coefficient and the reduction in the lattice thermal conductivity. As shown in Figure 8b, the average *ZT*s in the temperature range of 300–750 K of the Ti_1−*x*_Nb*_x_*(FeCoNi)Sb pseudo-ternary HH alloys were dozens of times that of TiCoSb, and this trend is further increased after the incorporation of Nb.

## 4. Conclusions

High-purity and high-density Ti_1−*x*_Nb*_x_*(FeCoNi)Sb (*x* = 0, 0.01, 0.03, 0.05, 0.07 and 0.1) pseudo-ternary half-Heusler alloys were successfully prepared via microwave synthesis combined with rapid hot-pressing sintering. The multi-element substitution at the Co sites can effectively reduce the lattice thermal conductivity by introducing more phonon scattering mechanisms. In addition, the lattice thermal conductivity was greatly reduced due to the increase in phonon scattering caused by point defects, mass fluctuation and strain fluctuation introduced by Nb doping. The average *ZT* values of the Nb-doped pseudo-ternary half-Heusler samples were dozens of times that of TiCoSb prepared using the same process. Of course, the factors affecting the electrical properties that are too different from other HH material systems also need to be further studied.

## Figures and Tables

**Table 1:** Room-temperature density (g/cm^3^), relative density (%), carrier concentration *n* (10^20^ cm^−3^) and carrier mobility *μ* (cm^2^v^−1^s^−1^) of TiCoSb and Ti_1−*x*_Nb*_x_*(FeCoNi)Sb (*x* = 0, 0.01, 0.03, 0.05, 0.07 and 0.1) alloys.

Composition	Density (g/cm^3^)	Relative Density (%)	Carrier Concentration (10^20^ cm^−3^)	Mobility (cm^2^v^−1^s^−1^)
TiCoSb	7.133	95.70	4.673	2.885
Ti(FeCoNi)Sb	7.104	95.30	1.471	5.914
Ti_0.99_Nb_0.01_(FeCoNi)Sb	7.093	95.16	1.562	5.451
Ti_0.97_Nb_0.03_(FeCoNi)Sb	7.184	96.38	1.866	4.873
Ti_0.95_Nb_0.05_(FeCoNi)Sb	7.254	97.32	2.185	4.467
Ti_0.93_Nb_0.07_(FeCoNi)Sb	7.315	98.13	2.454	4.239
Ti_0.90_Nb_0.10_(FeCoNi)Sb	7.331	98.39	2.787	3.966

**Table 2:** The phase fractions of Ti_1−*x*_Nb*_x_*(FeCoNi)Sb (*x* = 0, 0.01, 0.03, 0.05, 0.07 and 0.1) alloys.

Composition	HH (%)	Fe (%)	FeSb (%)
Ti(FeCoNi)Sb	95.1	4.9	~
Ti_0.99_Nb_0.01_(FeCoNi)Sb	94.6	5.4	~
Ti_0.97_Nb_0.03_(FeCoNi)Sb	92.8	7.2	~
Ti_0.95_Nb_0.05_(FeCoNi)Sb	90.3	9.7	~
Ti_0.93_Nb_0.07_(FeCoNi)Sb	84.5	11.3	4.2
Ti_0.90_Nb_0.10_(FeCoNi)Sb	79.1	13.6	7.3

## Data Availability

Not applicable.
